# Dexmedetomidine in Prevention of Postoperative Delirium: A Systematic Review

**DOI:** 10.7759/cureus.25639

**Published:** 2022-06-03

**Authors:** Jack Fondeur, Lisbeth Escudero Mendez, Mirra Srinivasan, Ranim K Hamouda, Baba Ambedkar, Hadia Arzoun, Isra Sahib, Lubna Mohammed

**Affiliations:** 1 Internal Medicine, California Institute of Behavioral Neurosciences & Psychology, Fairfield, USA; 2 Pathology, California Institute of Behavioral Neurosciences & Psychology, Fairfield, USA

**Keywords:** dexmedetomidine, delirium, prevention, cognitive dysfunction, postoperative delirium, anesthesiology

## Abstract

Delirium is defined by the DSM-5 as a fluctuating course of disturbance in attention, cognition, and awareness that develops over a short period without any pre-existing neurocognitive disorder. As people age, there is an increased risk of complications that may occur following a surgical procedure and one such acute complication is delirium. Studies are emerging to reduce the incidence of postoperative delirium, and one such preventive measures implemented in recent years include the administration of dexmedetomidine, a high selectivity α-2 adrenoceptor agonist. This study aims to review the efficacy of Dexmedetomidine in the prevention of postoperative delirium in randomized controlled trials in patients older than 18 years of age. The literature was explored in three online databases, namely, PubMed, Science Direct, and Scopus. Appropriate keywords and MesH terms were employed to scrutinize relevant articles that demonstrated the effects of dexmedetomidine in the prevention of postoperative delirium. The data was restricted to randomized controlled trials and clinical trials published from 2017 to 2021 in human patients older than >18 years of age undergoing non-cardiac-related procedures. The randomized clinical trials were critically assessed with the Cochrane risk of bias tool. We proceeded to screen 428 records with the assessment of the PRISMA chart and filtered out 420 papers to obtain a total of eight studies where we identified data such as sample size, types of surgeries in which the patients were involved, the delirium assessment tool, the plan of the administration of dexmedetomidine and the outcomes evaluated in each study. The Confusion Assessment Method (CAM) was the prevailing assessment tool used with the sole purpose to evaluate the incidence of postoperative delirium as the primary outcome, and assessment of inflammatory cytokines, sleep quality, and pain scales were considered as secondary outcomes. The dosage of dexmedetomidine varied among studies, and it displayed varying impacts on postoperative delirium and the secondary outcomes as well. Limitations include varying ages and ethnicities of the population. It was concluded that dexmedetomidine prevents the development of postoperative delirium in elderly patients undergoing non-cardiac surgical interventions by modulating important predisposing factors such as neuroinflammation, pain, and sleep quality. No funding was made for this study.

## Introduction and background

Delirium is defined by the Diagnostic and Statistical Manual of Mental Disorders, Fifth Edition (DSM-5) as a disturbance in attention, cognition, and/or awareness that develops over a short period, has a fluctuating course, and is accompanied by a change in cognition [1]. This condition affects, in particular, the elderly population, especially when predisposed to cognitive disorders, infections, or trauma [2]. As the human organism advances in age, physiologic changes occur, such as greater sensitivity to anesthetic drugs, higher drug concentrations at central nervous system receptors (SNC), and an increased risk of postoperative cognitive dysfunction and postoperative delirium (POD) [3]. The full mechanism behind POD remains unknown, but it’s suggested that it is a multifactorial condition influenced by neurotransmitter imbalance, neuroinflammation, brain network dysfunction, and endocrine stress response [4]. Delirium affects around 37% to 46% of the general surgical population. POD incidence can vary from 9% to 87%, depending on the type of surgery and the age of the patient [5]. POD has a higher prevalence in cardiovascular interventions and older adult patients [6]. Postoperative delirium has an onset of 24 to 72 hours after a surgical intervention but also has been linked with long-term cognitive dysfunction [7]. As evident, POD can result in a poorer quality outcome after surgical interventions. 

Dexmedetomidine (DEX) is a drug that acts as an α-2 adrenoceptor agonist with high selectivity, resulting in a blockage of the sympathetic nervous system that leads to sedation, hypnosis, and analgesia. This drug is used in the Intensive Care Unit (ICU) setting for postoperative sedation and some small invasive procedures [3]. Uses of DEX in the perioperative setting offer benefits such as anxiolysis decreased reactivity of the trachea at the intubation and extubating of the patient. Other benefits include extending the time of motor and sensory epidural blocks and demonstrating a synergism with other anesthetics with no morbidity affix [8]. On the other hand, the analgesic effect of this drug provides an advantage over other analgesics like opiates as it does not cause respiratory depression in the medullary centers. In addition, DEX has manifested less body movement in patients undergoing endoscopic procedures in comparison with midazolam administration [9].

The use of dexmedetomidine with the intention to prevent postoperative delirium remains controversial. Where it has shown to be effective in older adult patients undergoing cardiovascular and non-cardiovascular surgeries [10,11], as well in controlling hypoxemia, tachycardia, and hypertension [12]. It has resulted in not being effective in the administration of small doses in addition to propofol sedation with the purpose of decreasing POD [13]. Doses of DEX at 1 µg/kg have been revealed to control cough, agitation, hypertension, tachycardia, and shivering [14]; otherwise optimal dosing and timing for prevention of postoperative delirium remain unclear [12]. “In that sense, appropriate population and timing for prescribing DEX need to be further explored [8,15] to draw finite conclusions in preventing postoperative delirium.”

This study aims to analyze the efficacy of DEX in the prevention of postoperative delirium in adult patients undergoing non-cardiac surgical procedures imparting a better understanding of DEX to clinicians in an attempt to prevent the unfavorable complication of postoperative delirium.

Methods 

Three databases, PubMed, Science Direct, and Scopus, were explored online, employing the use of appropriate keywords and medical subject headings (MesH) terms to precisely get out all potentially relevant articles demonstrating the effect of dexmedetomidine in the prevention of postoperative delirium. The Boolean scheme was used to galvanize the keywords and the MeSH strategy format and subsequently employed in PubMed. The keywords used include anesthesiology, postoperative delirium, cognitive dysfunction, prevention, delirium, dexmedetomidine. 

Inclusion and exclusion criteria

The study was restricted to randomized control trials and clinical trials published during 2017-2021. In the studies selected, it was ensured all of them were free full articles, were conducted on humans only and that all patients were at least 18 years of age. Patients undergoing cardiovascular surgical procedures were not included. In addition, only studies in English were included. The population, intervention, comparison, outcomes, and study criteria (PICOS) were the basis for the criteria selection.

Data collection

Data selection and extraction were carried out independently by two researchers (FJ and EL). In cases of disagreements, both researchers discussed the study design, inclusion and exclusion criteria, and intervention. In instances when neither of the two researchers agreed, an opinion of a third reviewer was requested.

Analysis of study quality 

**Figure 1:**
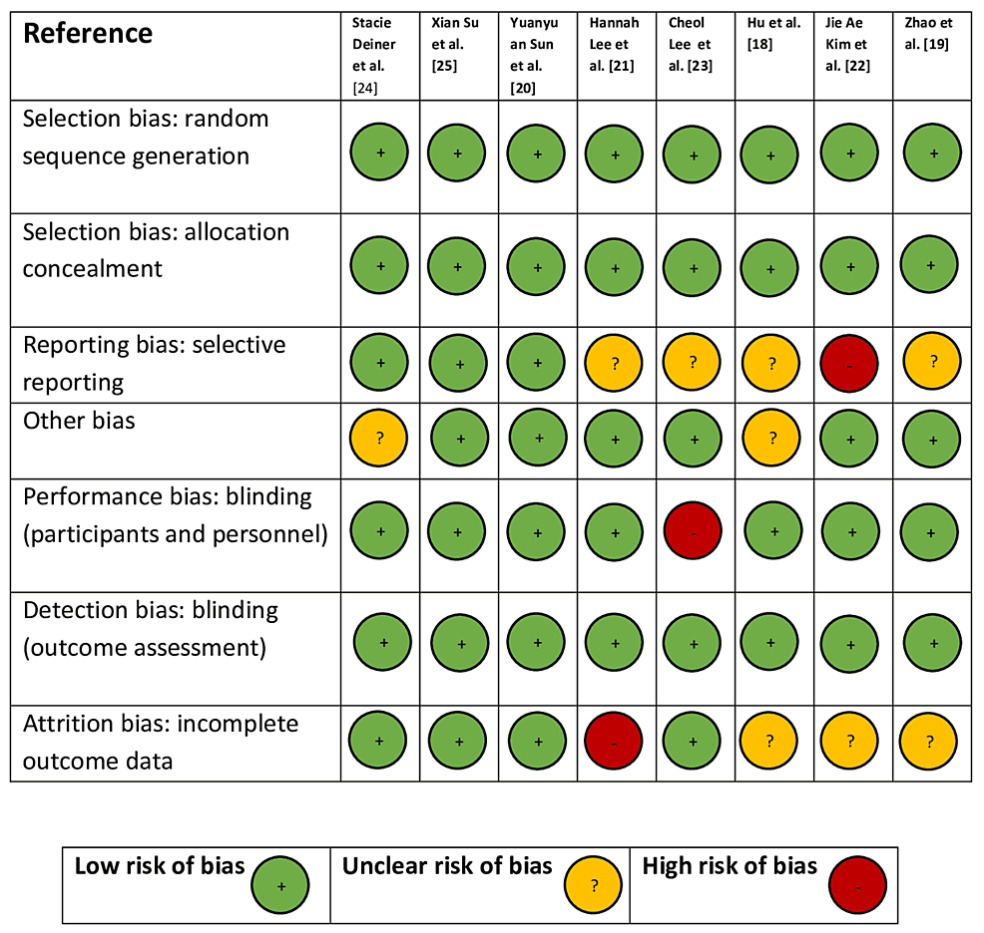
Cochrane risk of bias tool for included studies Source: The Cochrane Collaboration’s tool for assessing risk of bias in randomised trials [16].

The randomized clinical trials were critically assessed with the Cochrane risk of bias tool; each study was probe based on the seven criteria to exclude potential biases. Each criterion was scored as either high risk, low risk, or unclear. A brief summary of the tool is given in Figure 1.

Managing records

This search was made from 1^st^ to 18^th^ September 2021, at the end of this date, the total of records was 325 from all three databases. Duplicates were eliminated in a Microsoft Excel sheet (Redmond, USA). 

Results

**Figure 2:**
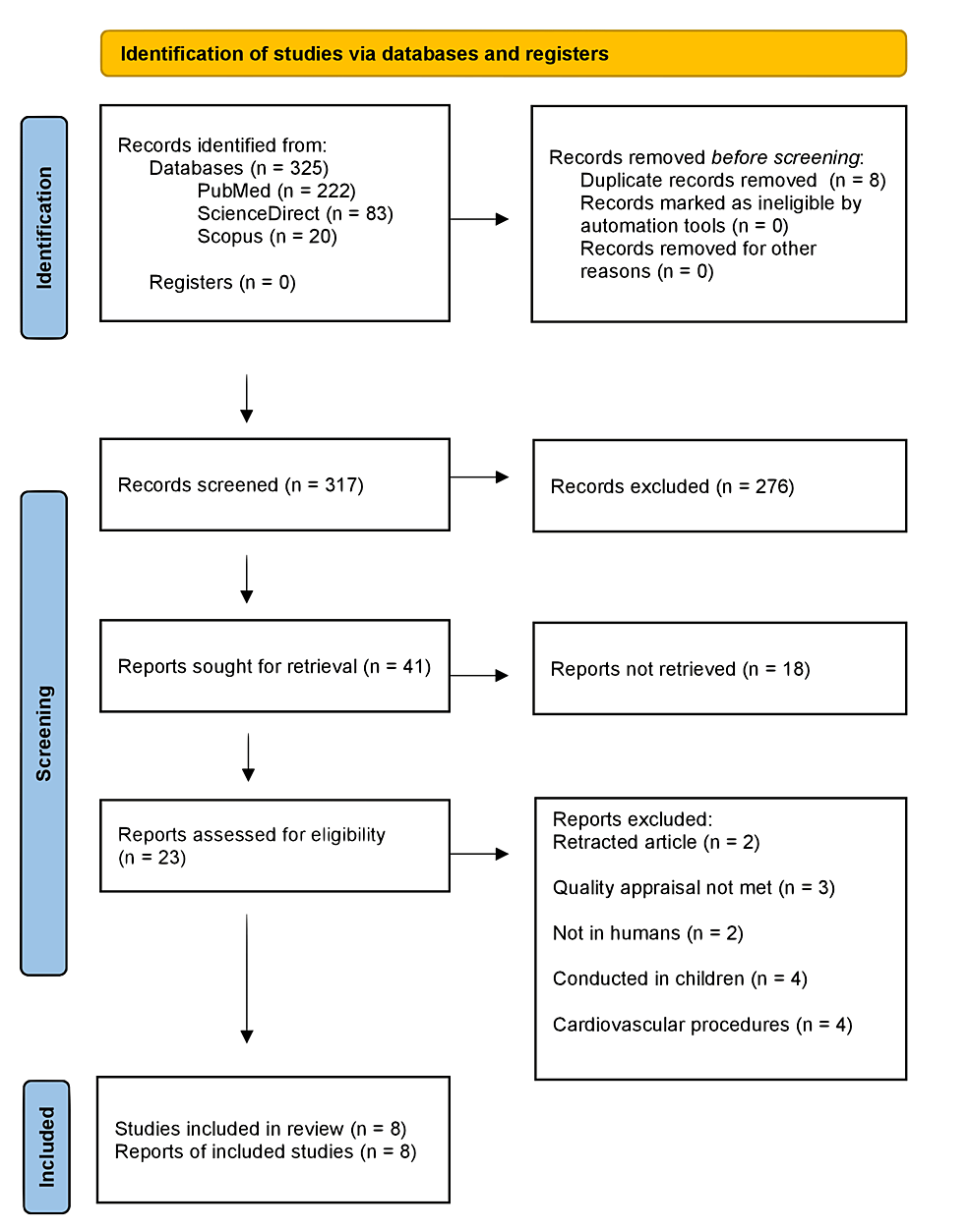
PRISMA chart for identification of studies via databases Source: The PRISMA 2020 statement: an updated guideline for reporting systematic reviews [17]

The identification of studies via databases and registers in accordance with Preferred Reporting Items for Systematic Reviews and Meta (PRISMA) 2020 guidelines [16]. The total records identified were 325 studies collected through PubMed, ScienceDirect, and Scopus databases. Of which eight duplicate studies were removed through a Microsoft Excel sheet, 276 records were excluded based on the inclusion and exclusion criteria of the study. A total of 18 studies were not retrieved, with 23 assessed for eligibility and 15 excluded for different causes. A total of eight studies were included in this review. The PRISMA flow diagram and the search process followed are illustrated in Figure 2.

After identifying the included articles, it proceeded to summarize relevant information such as sample size, types of surgeries in which the patients were involved, the delirium assessment tool, the plan of the administration of dexmedetomidine, and the outcomes evaluated in each study. This is described altogether in Table 1 [18-25].

**Table 1:** Summary of identified articles DEX: Dexmedetomidine, CAM: Confusion assessment method, CAM-ICU: Confusion assessment method for the intensive care unit

Author	Year	Mean age/age (yr)	Sample size (n)	Type of surgeries	Delirium assesment	DEX administration	Outcomes
Hu et al. [18]	2021	70	177	Transthoracic oesophagectomy	CAM	Loading dose of DEX (0.4 mg kg), over 15 min, followed by a continuous infusion at a rate of 0.1 mg/kg/h and 1hr post-surgery	Delirium incidence, the incidence of emergence agitation, serum interleukin-6 (IL-6) levels, and haemodynamic profile
Zhao et al. [19]	2020	69	432	Thoracic, general, genitourinary, gynecologic, and orthopedic	CAM	DEX 0, 100, 200, and 400 μg with sufentanil 150 μg for PCA immediately after surgery	Delirium incidence, nausea, vomiting, dizziness, headache, sleepiness, bradycardia, hypotension, and respiratory depression
Sun et al. [20]	2019	68.5	557	Spine; orthopedic; urologic; thoracic; and general surgery	CAM and CAM-ICU	IV DEX (dexmedetomedine 0.1 μg/kg/h) post-surgery with continuous infusion for 48 hours	Delirium incidence, pain scoring at rest and movement, supplemental analgesics, subjective sleep quality
Lee et al. [21]	2019	56	201	Living-Donor Liver Transplantation	CAM-ICU	Infusion DEX (0.1mcg/kg/hour) immediately after induction of anesthesia and continued 48 hours postoperatively	Delirium incidence, duration of delirium, mechanical ventilation duration, ICU length of stay, hospital length of stay, use of antipsychotics and sedatives during ICU stay, post-ICU psychiatric consultation for delirium, in-hospital mortality, and mortality at three months
Kim et al. [22]	2019	65	143	Thoracic surgery	CAM and CAM-ICU	IV DEX started after inducing anesthesia and continued until the end of surgery at a fixed dose (0.5 μg/kg/hr)	Delirium incidence, pro-and anti-inflammatory cytokines, and catecholamines
Lee et al. [23]	2018	73	354	Laparoscopic or robotic assisted radical cystectomy; laparoscopic or robotic assisted partial or total nephrectomy; laparoscopic or robotic assisted colorectal surgery	CAM	Group 1: 1 μg/kg bolus followed by 0.2–0.7 μg/kg/h in- fusion from induction of anesthesia to the end of surgery Group 2: 1 μg/kg diluted to a total volume of 10mL in saline [0.9%] over a 10 min period 15 min before the end of surgery	Delirium incidence, duration of delirium for five days after surgery, cortisol, C-reactive protein (CRP), and cytokine (tumor necrosis factor [TNF]-alpha, interleukin [IL]-1β, IL-2, IL-6, IL-8, and IL-10) levels
Deiner et al. [24]	2017	74	390	Spine, thoracic, orthopedic, urologic, and general surgery	CAM, CAM-ICU.	DEX infusion (0.5µg/kg/h) intraoperative and continued 2 hours after surgery	Delirium incidence, bradycardia, hypotension, hypertension, serious adverse effects, mortality, and length of stay
Su et al. [25]	2016	> 65	700	Intra-abdominal; intra-thoracic; spinal and extremital; superficial and transurethral	CAM-ICU	IV DEX infusion (0.1 μg/kg/h) after patients were post-surgery in the ICU until the next day	Delirium incidence, time to extubation, overall incidence of non-delirium complications, length of stay in ICU, length of stay in hospital after surgery, and all-cause 30-day mortality

## Review

After a search of all the relevant reports, this study is mainly focused on discussing predisposing factors that could increase the incidence of postoperative delirium, the point of reference in delirium assessment, and on what basis is a plan constructed in administering the dexmedetomidine.

Predisposing factors for postoperative delirium

Surgical procedures and anesthesia have been associated with an increase in cytokines, such as IL-6 and TNF-alpha, contributing to the development of neuroinflammation that can predispose patients to conditions like cognitive impairment after interventions [26]. Therefore, the drugs used in general anesthesia increase the incidence of developing delirium by altering the chemical balance in the sleep/arousal pathway [27-28]. Dexmedetomidine has proven to decrease the levels of IL-6 cytokines in patients induced with total intravenous anesthesia [18]. In addition to IL-6, DEX also has been reported to be effective at reducing levels of cortisol, C-reactive protein, [TNF]-alpha, and other interleukins such as IL-1β levels at one hour and 24 hours after surgery [23].

Advanced age, especially over 65 years old, among others, is a strong risk factor for developing postoperative delirium [29]. In the studies reviewed, the most prevalent population was well over 65 years of age, except for one study with a median age of 56 years [21]. On the other hand, low preoperative cognitive function is another important risk factor for developing POD [29]. The same was evidenced in patients who have not completed certain levels of education, such as a high school with a baseline Mild Cognitive Impairment test, therefore predisposing the patient to develop POD [24]. 

Pain is another risk factor that can be present in the pre-operative and post-operative setting, increasing the risk for the development of POD. Preoperative pain, as well as depression, have been evaluated and associated with a higher incidence of developing POD [30,18]. Nonetheless, acute postoperative pain through C-reactive protein marker has been evidenced to increase the risk of developing POD [31-32]. Therefore, intentions to manage pain with opioids increase the risk for the development of POD [33]. Pain has been assessed through the Numeric Rating Scale (NRS, an 11-point scale where 0 indicated no pain and 10 indicated the worst possible pain) [34] and showed no increased risk associated with POD, alternatively, the group managed with DEX demonstrated improved scores in the NRS scale in contrast with the placebo group [20]. Similarly, the use of DEX reduced the administration of propofol and opioids, maintaining similar anesthesia Bispectral index (BIS) levels in patients managed with total intravenous anesthesia, although the researchers didn’t evaluate pain with an authentic scale. 

Delirium assessment 

To facilitate the identification of delirium screening can be assessed by many tools such as the Confusion Assessment Method (CAM), Richmond Agitation-Sedation Scale (RASS), Memorial Delirium Assessment Scale (MDAS) and the Delirium Rating Scale-Revised-98 (DRS-R98) and Delirium Screening Checklist (ICDSC) [35-36]. The Confusion Assessment Method (CAM) evaluates four features: (a) an acute onset and fluctuating course of mental state, (b) inattention, (c) disorganized thinking, and (d) an altered level of consciousness. Variants of this tool, like the CAM-ICU, provide a two-minute evaluation that can be addressed in the intensive care unit with a 93% accuracy. The Richmond Agitation-Sedation Scale (RASS) tool evaluates the level of sedation or alertness through a numerical value range of -5 to +4 [28].

Of all the tools, the RASS and CAM were the ones that the studies utilized to assess delirium. In particular, all eight studies assessed delirium with either the CAM or its variant CAM-ICU [18-25]. This tool has been proven to be the most widely used for the identification of delirium in clinical research and practice [37]. On the other hand, the RASS scale was used to assess sedation and served as a further classification for delirium, those being: hypoactive, hyperactive, and mixed [25]. In other cases, the RASS scale was used to identify if the patient was unarousable or deeply sedated; from there just, if the patient had a score of more than -3, a delirium assessment was performed [20,21].

Delirium assessment with the CAM tool was addressed differently among the studies reviewed, ranging from one to three times a day for a different number of days after the surgical interventions. However, the primary method of the evaluation for POD was twice a day for at least five days postoperatively [18,20,23,25]. Other studies integrated additional information about delirium, such as the onset [19-21,23,25], duration [19,21,23], psychiatrist assistance [21,23], pain [19-20,22-23,25] and sleep quality with subjective scales [20,23,25]. According to suggestions as per the postoperative prevention guidelines, pain and sleep quality are the crucial measurements in managing POD [38].

Dexmedetomidine administration 

The most frequent dose administered to patients to modify the incidence of postoperative delirium in an intravenous infusion was 0.1 μg/kg/h; the timing for administration was immediately after induction [21] and leaving an infusion after surgery for either the next day [25] or a period of 48 hours [21]. However, in just one of these trials the incidence of delirium was significantly lower with 9.1% vs. 22.6% of those who received a placebo (p=0.001) [25]. Moreover, DEX showed to be efficient at decreasing POD administered at a loading dose of 0.4 mg/kg over 15 minutes, preceded by a continuous infusion of 0.1 mg/kg/h during the intervention and after one hour after surgery [18]. Interventions that had more than one study group with a different dose of DEX obtained a positive decrease in POD incidence [19,23]. The duration time of delirium was not significantly different in groups with a dose of 1μg/kg/ bolus followed by 0.2-0.7 μg/kg/h infusion from the time of induction to the end of anesthesia from patients given a 1 μg/kg bolus 15 minutes before the end of the surgical intervention. However, the incidence of delirium was reduced to half in patients who had DEX administration of 1μg/kg/ bolus followed by 0.2-0.7 μg/kg/h infusion from the time of induction to the end of surgery (p=0.017) [23]. Intraoperative infusion doses of (0.5 μg/kg/hr) were demonstrated to not affect the decreasing incidence of POD in two studies, with either prolonged administration after surgery or stopping the infusion at the end of the surgery [22,24].

Multiple dosing with 0, 100, 200, and 400 μg of DEX co-administered with 150 μg of sufentanil on the postsurgical setting demonstrated a dose-effect on decreasing both POD and postoperative cognitive dysfunction recorded at days one, two, three, and seven after surgery (p<0.05). Duration and onset were also found to be decreased in patients over 70 years old, with no major difference with doses of 200 μg compared to 400 μg (p=0.027). The incidences of postoperative nausea, vomiting, dizziness, headache, and sleepiness demonstrated no significant differences among all groups in the recorded days [19].

Furthermore, DEX has exhibited sleep-improving effects in critically ill patients on increasing stage 2 and overall sleep efficiency [39]. Similar findings were found in the analyzed studies that evaluated the subjective sleep quality with improved scores in patients who received DEX doses of 0.1 μg/kg/h after surgery for 48 hours [20,25]. The administration of DEX has also been proven to have an effect, improving the score in the NRS pain scale (p<0.05) [20,21,25] and the visual analog scale (VAS) [23]; although measured by the VAS, it showed no improvement on the groups that received doses of 0, 100, 200, and 400 μg co-administered with 150 μg of sufentanil [19]. 

The most prevalent complication postoperatively is pain; therefore, management with opioids could be beneficial. However, opioids tend to worsen postoperative sleep by decreasing the rapid eye movement (REM) cycle and thus stimulating arousal. Another postoperative complication with the same propensity as pain-causing POD is sleep disturbances in patients undergoing non-cardiac surgery [40]. As a result, preventing these complications with DEX can benefit pain management by reducing the need for additional pain medications such as opioids and inhalant agents while also optimizing the sleep quality and consequently decreasing the overall likelihood of developing postoperative delirium.

**Figure 3:**
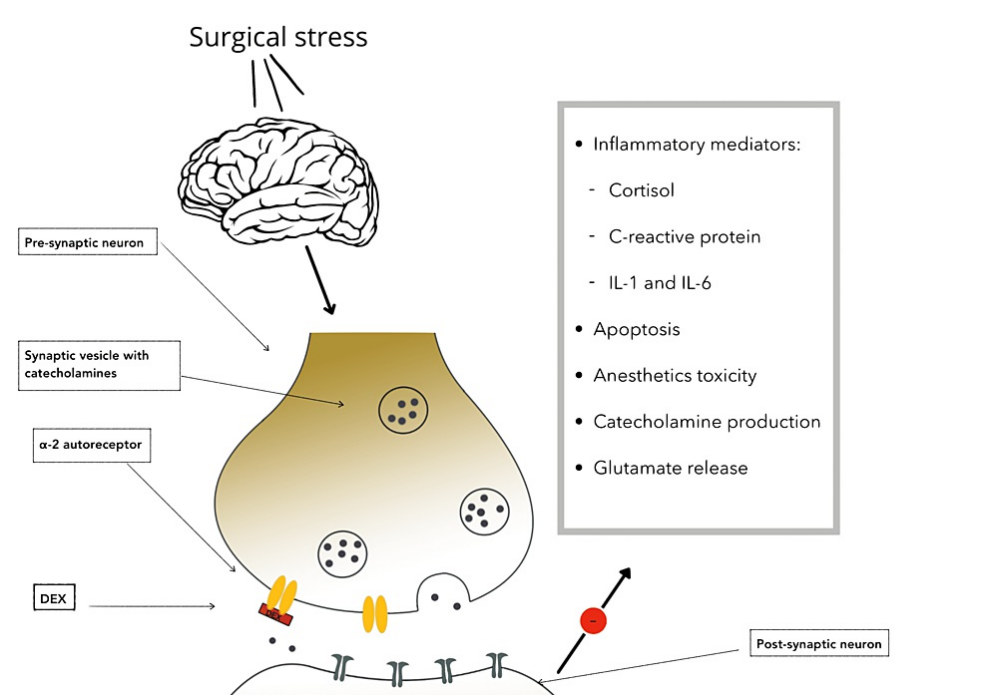
Dexmedetomidine mechanism of action Image created in the Sketchbook app for iOS. DEX: dexmedetomidine, L-1: Interleukin-1, IL-6: Interleukin-6, α2: alpha-2

In addition to this, dexmedetomidine has also been proven to modulate inflammation in a multiorgan fashion, including inflammation across the lungs, the heart, the liver, the kidneys, the small intestine, and the nervous system. The mechanism behind this is the decreased excitability by the sympathetic nervous system, modulating glutamate release, decreasing the toxicity of anesthetic drugs, inhibiting apoptosis, inflammatory cytokines, and regulation of neuronal plasticity [41,42]. With this, the DEX administration might not only decrease postoperative delirium condition but also other benefits that have a positive impact on the nervous system in a postsurgical condition and the stress that the body is exposed to. These mechanisms contribute to the decrease in the neuronal inflammation, neurotransmitter imbalance, and endocrine stress response suggested for developing postoperative delirium [4], which is better illustrated in Figure 3.

Limitations

This study has several limitations. The search was restricted to free, complete randomized control trials published from 2017 through 2021 conducted on humans and a population above 18 years of age. Studies performed on children and patients undergoing cardiac surgery were not evaluated. Another limitation of this study was that the majority of the trials were conducted in patients above 65 years of age, and data in patients younger than 54 couldn't be evaluated. Also, the majority of the studies included were conducted in the Asian population, and no extended evaluation was possible in other ethnicities. On the other hand, this study included randomized controlled trials only; thus, reviewing other types of studies could collect more data about the topic. 

## Conclusions

In sum, the administration of dexmedetomidine could prevent the development of postoperative delirium in adult patients above 65 years of age undergoing non-cardiac surgical interventions. By modulating important predisposing factors such as neuroinflammation via lowering levels of Tumor Necrosis Factor-alpha, Interleukin 6, and other proinflammatory cytokines at one and 24 hours post-surgery. Other benefits of the drug on the nervous system were decreased toxicity of anesthetic drugs and regulation of neuronal plasticity.

Moreover, postoperative pain was improved by the administration of dexmedetomidine by reducing the C-reactive protein levels and the Numeric Rating Score. Additionally, reducing the requirements for opioids while maintaining similar anesthesia depth levels. Further, sleep quality has been demonstrated to be improved by dexmedetomidine administration by increasing stage two and overall sleep efficiency. The most efficient administration of the drug seems to be a loading dose before surgery followed by a continuous infusion through the surgery and a few hours postoperatively. Further research is needed in larger populations with homogenous variables with respect to age, gender, and race besides assessing other measurements such as sleep quality and pain that are deemed essential. Future research could include evaluating history for cognitive impairment and assessing any abnormalities, including monitoring postoperative delirium at least twice a day, and obtaining psychiatric support, if available.
